# Supplementary material for: Network dynamics of momentary affect states and future course of psychopathology in adolescents
Source: PLoS One. 2021 Mar 4;16(3):e0247458. doi: 10.1371/journal.pone.0247458 (PMC7932519; doi:10.1371/journal.pone.0247458)
Supplement: S1 Table — (DOCX) [file pone.0247458.s001.docx]

S1 Table. *The network connections between ESM variables based on the B-coefficients from the autoregressive multilevel regression models and 95% confidence intervals for B-coefficients*

| The Stable group | | | | | | | | | | | | | |
| --- | --- | --- | --- | --- | --- | --- | --- | --- | --- | --- | --- | --- | --- |
| From |  | To | | | | | | | | | | | |
|  |  | *Cheerful* | | *Relaxed* | | Energetic | | *Irritated* | | *Down* | | *Lonely* | |
|  |  | *B* | *CI* | *B* | *CI* | *B* | *CI* | *B* | *CI* | *B* | *CI* | *B* | *CI* |
|  | Cheerful | .16* | .10-.21 | .10* | .04-.16 | .07* | .02-.11 | -.04 | -.09-.01 | -.06* | -.10-(-.02) | -.03 | -.08-.02 |
|  | *Relaxed* | .01 | -.02-.07 | .07* | .01-.13 | .04 | -.00-.08 | .02 | -.03-.07 | -.03 | -.07-.01 | -.00 | -.05-.04 |
|  | Energetic | .07* | .05-.17 | .06* | .01-.13 | .22* | .16-.28 | -.03 | -.09-.02 | -.04 | -.09-.00 | -.00 | -.06-.05 |
|  | Irritated | -.04 | -.06-.03 | -.01 | -.06-.03 | .00 | -.04-.05 | .10* | .05-.16 | .04 | -.00-.08 | .02 | -.02-.06 |
|  | Down | -.06 | -.08-.05 | -.00 | -.05-.05 | -.04 | -.10-.02 | -.01 | -.07-.06 | -.06 | -.00-.12 | .05 | -.00-.11 |
|  | Lonely | -.03 | -.07-.03 | -.04 | -.08-.01 | .01 | -.04-.05 | -.01 | -.06-.04 | .00 | .00-.07 | .05 | -.00-.11 |
| The Increase group | | | | | | | | | | | | | |
| From |  | To | | | | | | | | | | | |
|  |  | *Cheerful* | | *Relaxed* | | *Energetic* | | *Irritated* | | *Down* | | *Lonely* | |
|  |  | *B* | *CI* | *B* | *CI* | *B* | *CI* | *B* | *CI* | *B* | *CI* | *B* | *CI* |
|  | Cheerful | .12* | *.06-.19* | .05 | *-.00-.10* | .07* | *.02-.11* | .03 | -.02-.08 | -.00 | -.04-.04 | -.03 | -.07-.02 |
|  | *Relaxed* | .05* | *.00-.10* | .03 | *-.03-.09* | .03 | *-.02-.08* | -.01 | -.07-.05 | -.01 | -.04-.03 | -.00 | -.05-.04 |
|  | Energetic | .15* | *.09-.20* | .07* | *.02-.13* | .13* | *.08-.19* | -.09* | -.15-(-.03) | -.03 | -.08-.02 | -.02 | -.07-.03 |
|  | Irritated | -.00 | *-.04-.04* | -.01 | *-.05-.04* | -.01 | *-.05-.03* | .09* | .03-.15 | . 06* | .02-.09 | .01 | -.03-.04 |
|  | Down | -.05 | *-.11-.01* | -.00 | *-.07-.06* | -.04 | *-.10-.01* | .05 | -.03-.12 | .13* | .08-.19 | .11* | .06-.16 |
|  | Lonely | -.03 | *-.08-.02* | -.04 | *-.10-.01* | -.02 | *-.08-.03* | -.01 | -.06-.05 | .08* | .06-.13 | .10* | .03-.16 |

*Note. * - p-value was smaller than .05*
