# Supplementary material for: Network dynamics of momentary affect states and future course of psychopathology in adolescents
Source: PLoS One. 2021 Mar 4;16(3):e0247458. doi: 10.1371/journal.pone.0247458 (PMC7932519; doi:10.1371/journal.pone.0247458)
Supplement: S2 Table — (DOCX) [file pone.0247458.s002.docx]

### S2 Table. *Results of the permutation test*

| Measure | The Stable Group | The Increase Group | Group difference | | P-value |
| --- | --- | --- | --- | --- | --- |
|  |  |  | ***Diff.*** | ***%*** |  |
| Negative connectivity | .17 | .34 | .18 | 207 | .43 |
| Positive on negative | .24 | .15 | .09 | 159 | .67 |
| Negative on positive | .16 | .22 | .06 | 132 | .82 |
| Out-strength Cheerful | .33 | .17 | .16 | 197 | .28 |
| Out-strength Relaxed | .1 | .12 | .02 | 120 | .72 |
| Out-strength Energetic | .26 | .35 | .09 | 133 | .58 |

Note: “negative connectivity” refers to the strength of the network connections between negative affect states; “positive on negative” and “negative on positive” refers to the overall effect of the positive states (‘cheerful’, ‘relaxed’, ‘energetic’) on the negative states (‘irritated’, ‘down’, ‘lonely’) and vice-versa;
