# Supplementary material for: Network dynamics of momentary affect states and future course of psychopathology in adolescents
Source: PLoS One. 2021 Mar 4;16(3):e0247458. doi: 10.1371/journal.pone.0247458 (PMC7932519; doi:10.1371/journal.pone.0247458)
Supplement: S3 Table — (DOCX) [file pone.0247458.s003.docx]

S3 Table. All possible networks of “stable” and “increase groups” and characteristics of the “stable” groups.

| Networks | Vicious cycle (negative loop) in the stable group | Negative cluster in stable group, n of nodes | N of downregulating paths from positive cluster to negative one/negative nodes | |
| --- | --- | --- | --- | --- |
|  |  |  | Stable group | Increase group |
| 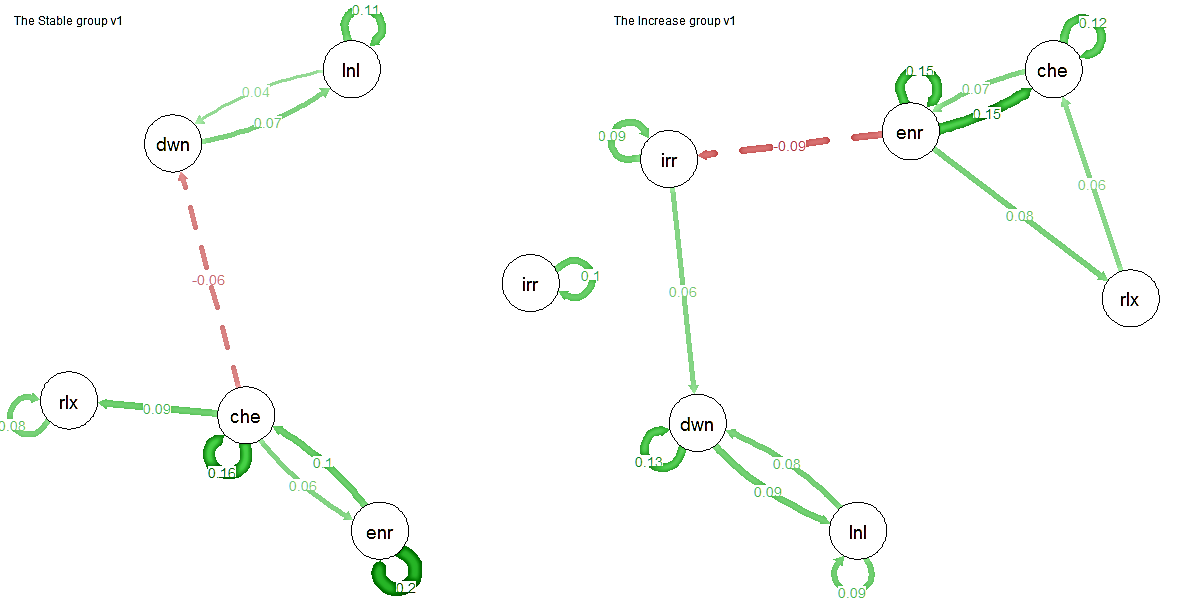 | 1 | 2 | 1 | 1 |
| 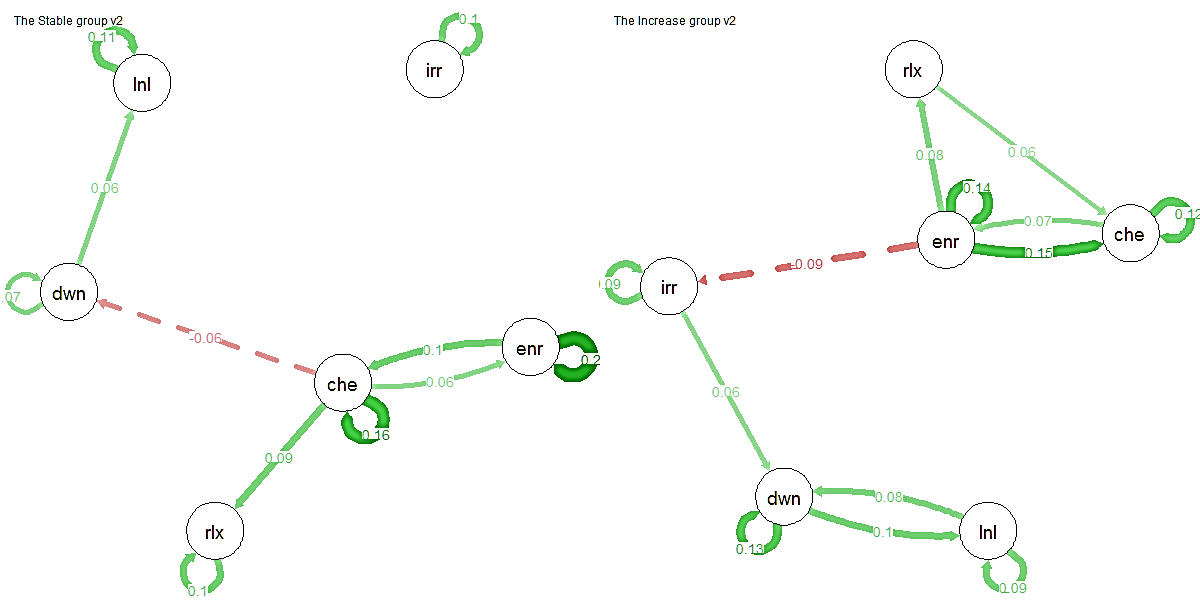 | 0 | 2 | 1 | 1 |
| 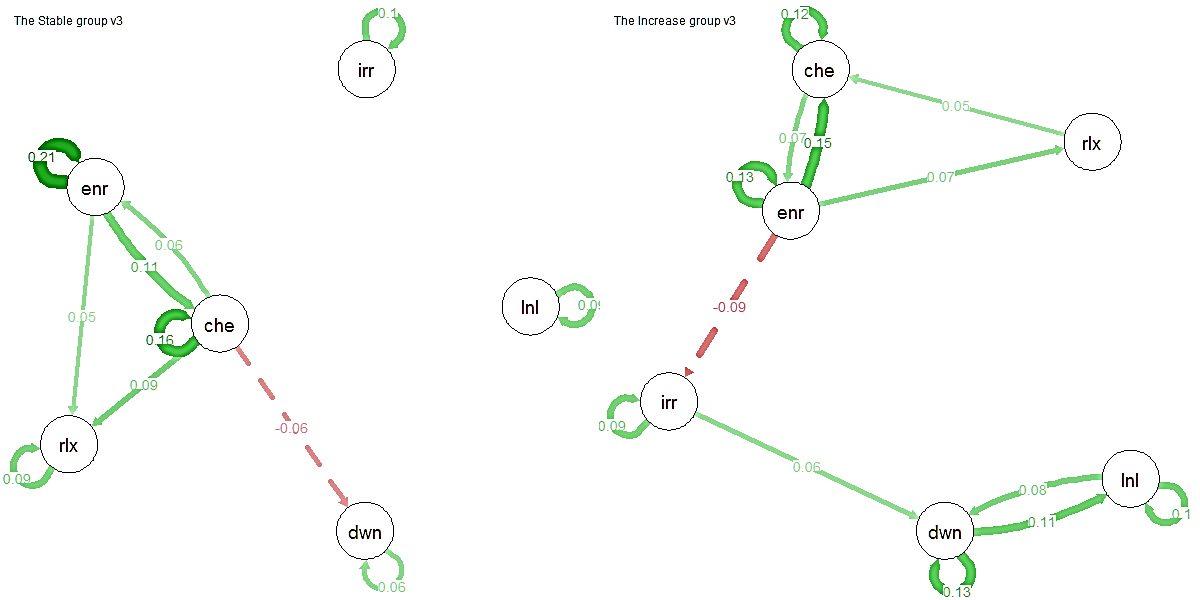 | 0 | 0 | 1 | 1 |
| 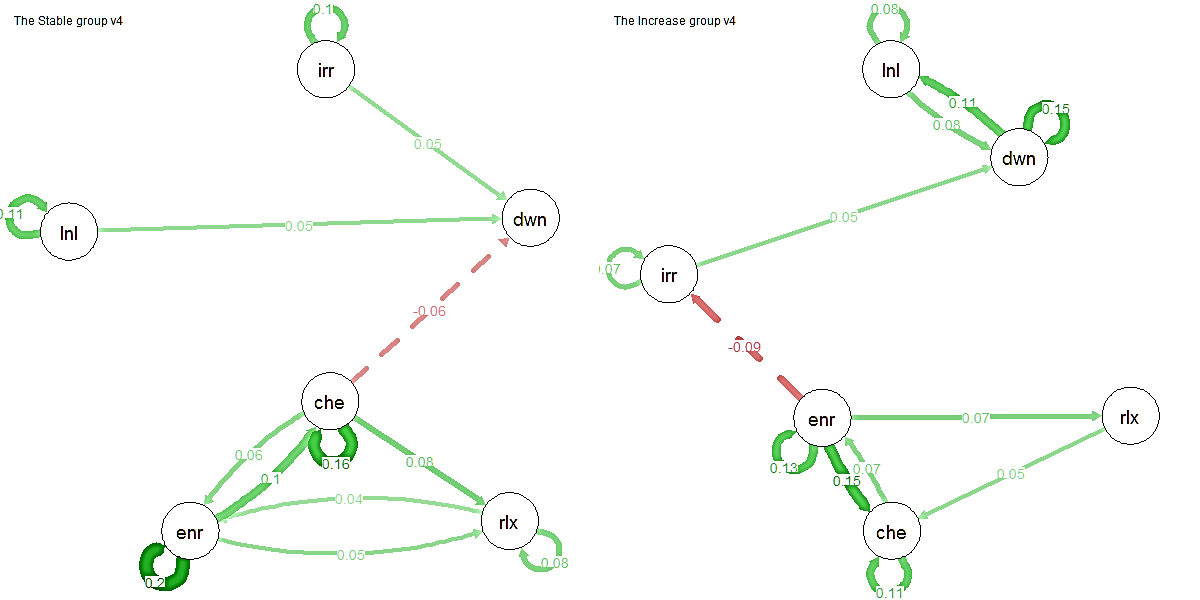 | 0 | 3 | 1 | 1 |
| 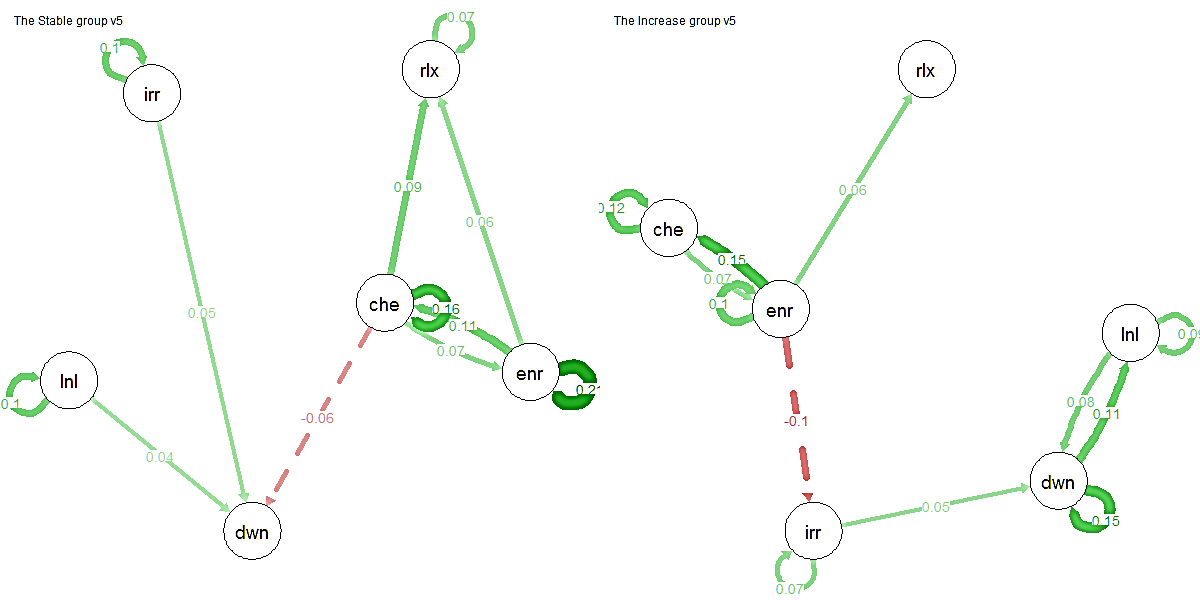 | 0 | 3 | 1 | 1 |
| 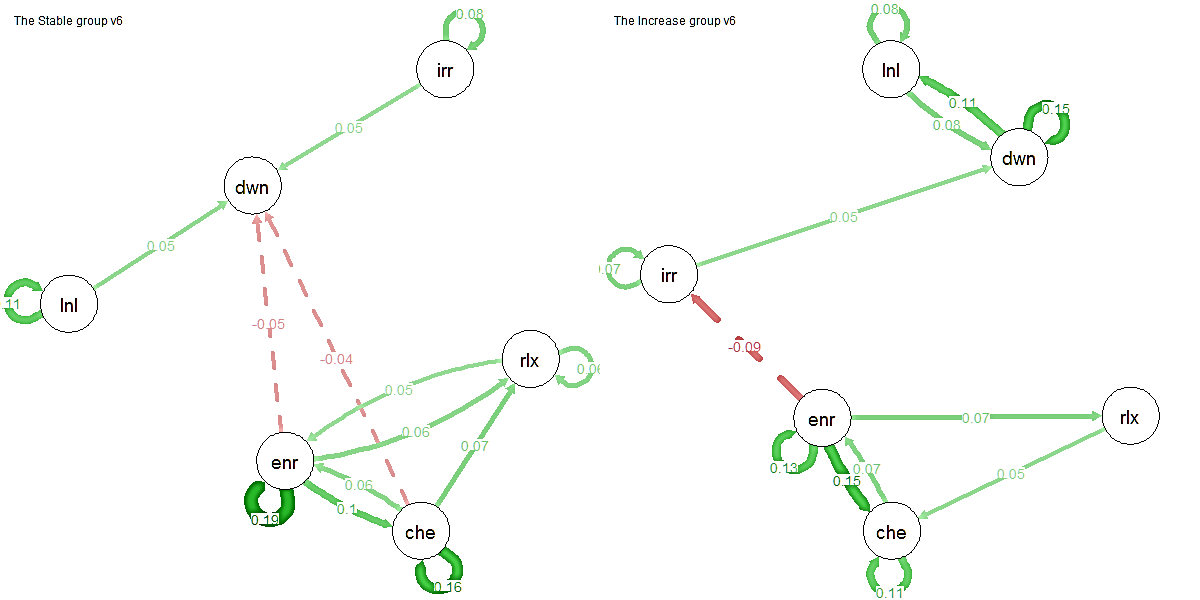 | 0 | 3 | 2 | 1 |
| 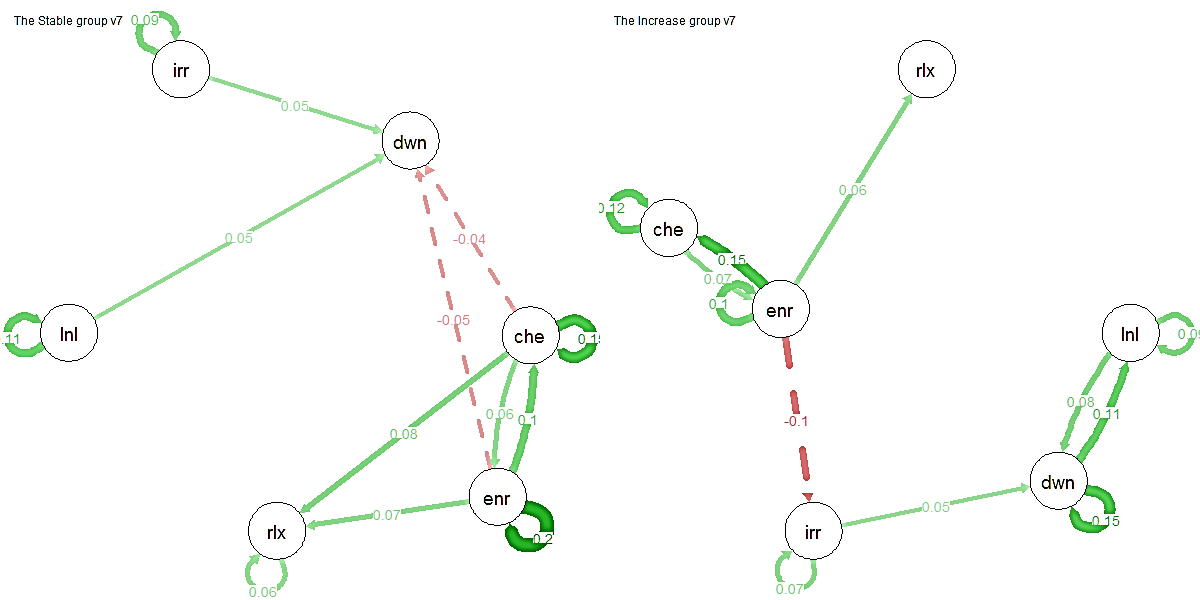 | 0 | 3 | 2 | 1 |
| 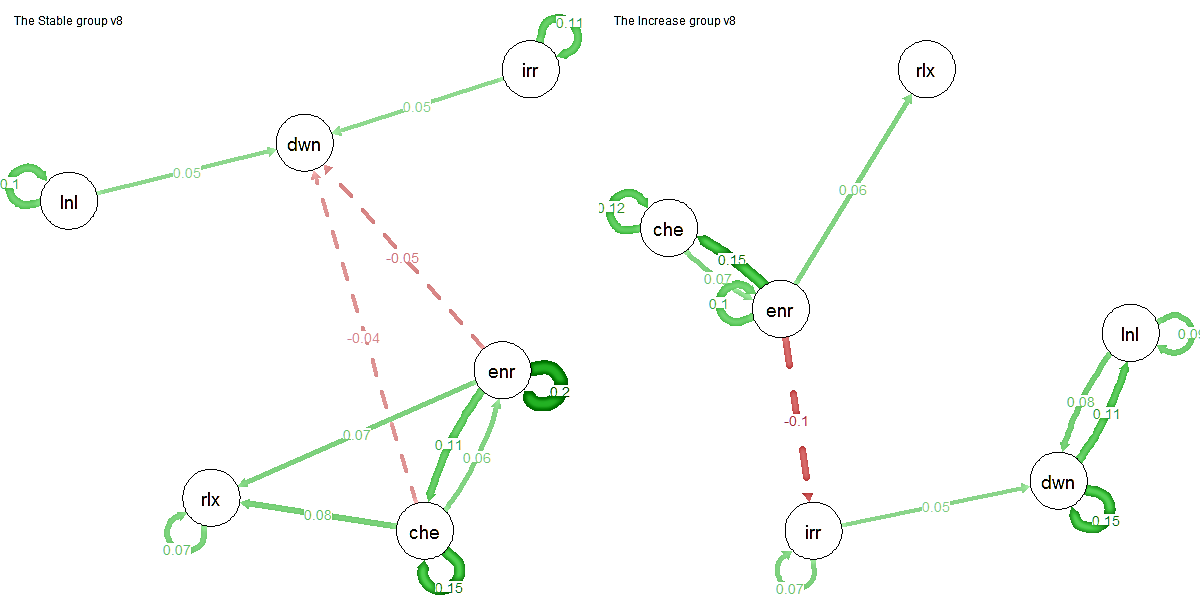 | 0 | 3 | 2 | 1 |
| 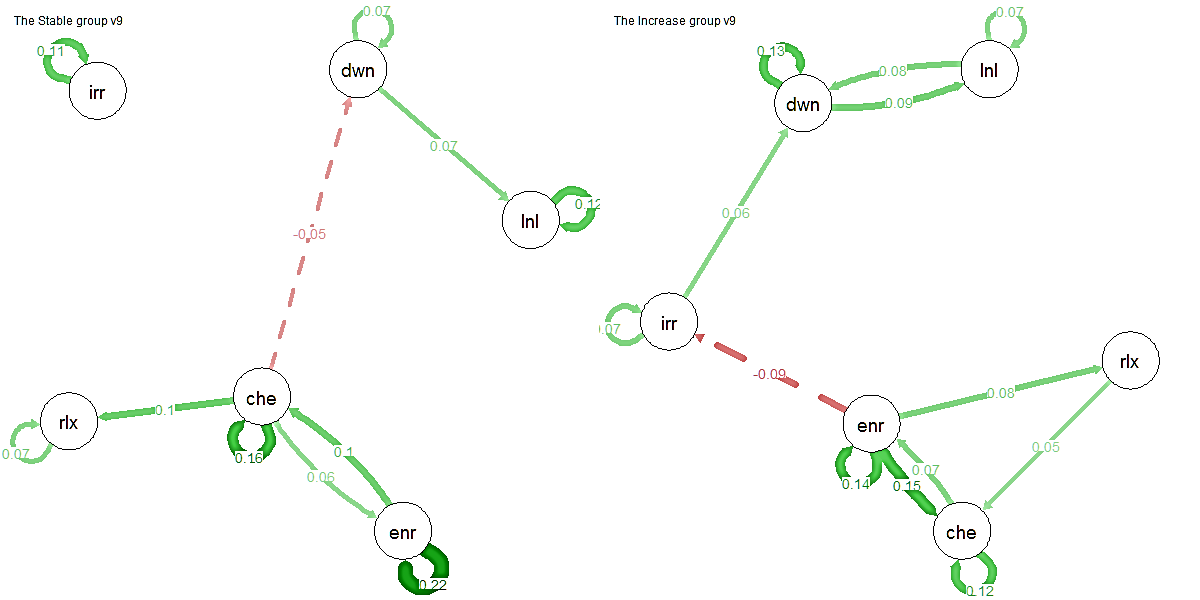 | 0 | 2 | 1 | 1 |
| 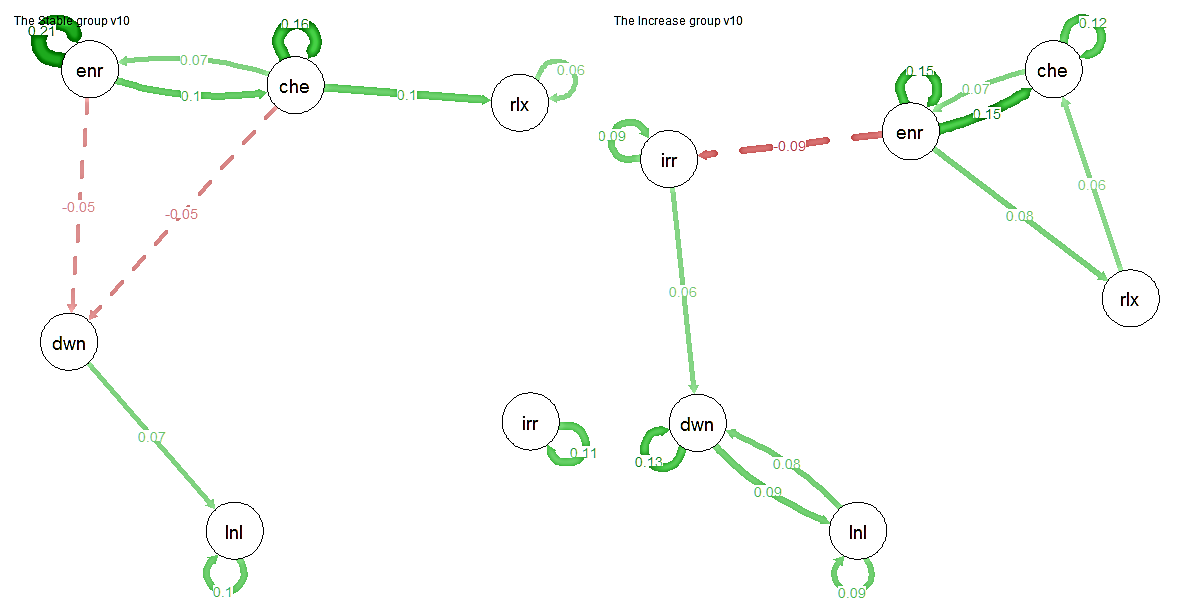 | 0 | 2 | 2 | 1 |
| 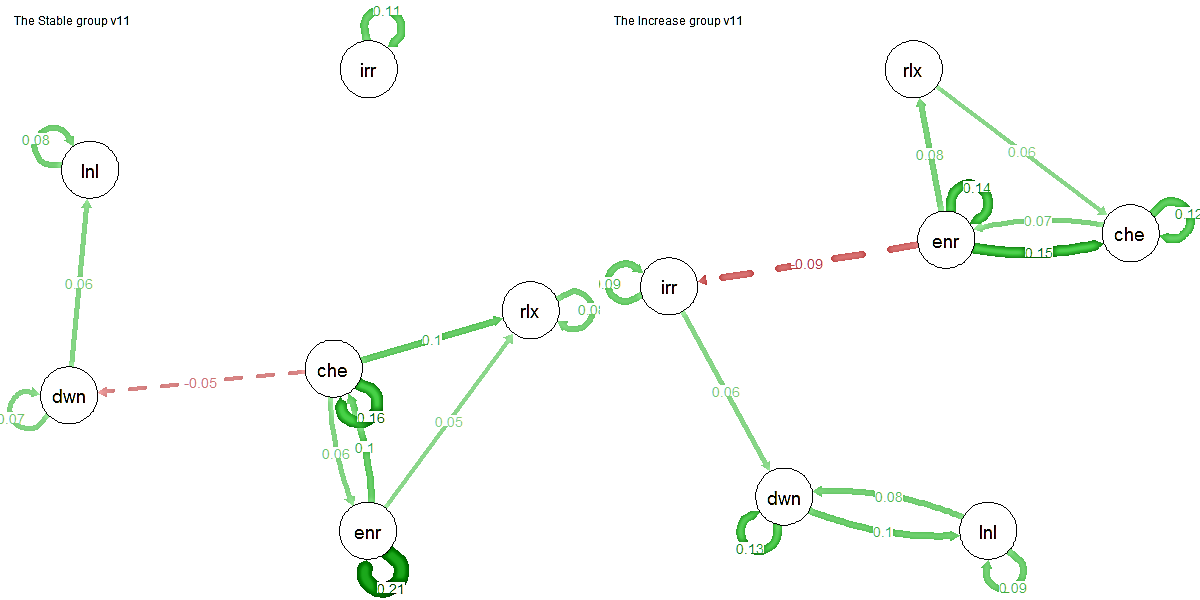 | 0 | 2 | 1 | 1 |
| 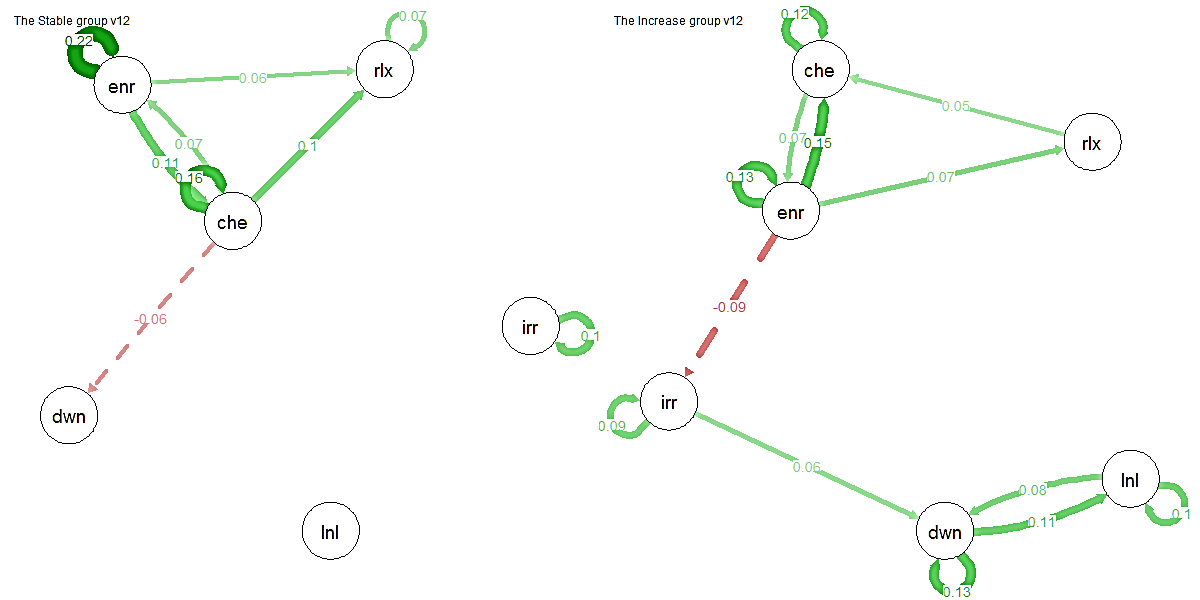 | 0 | 0 | 1 | 1 |
| 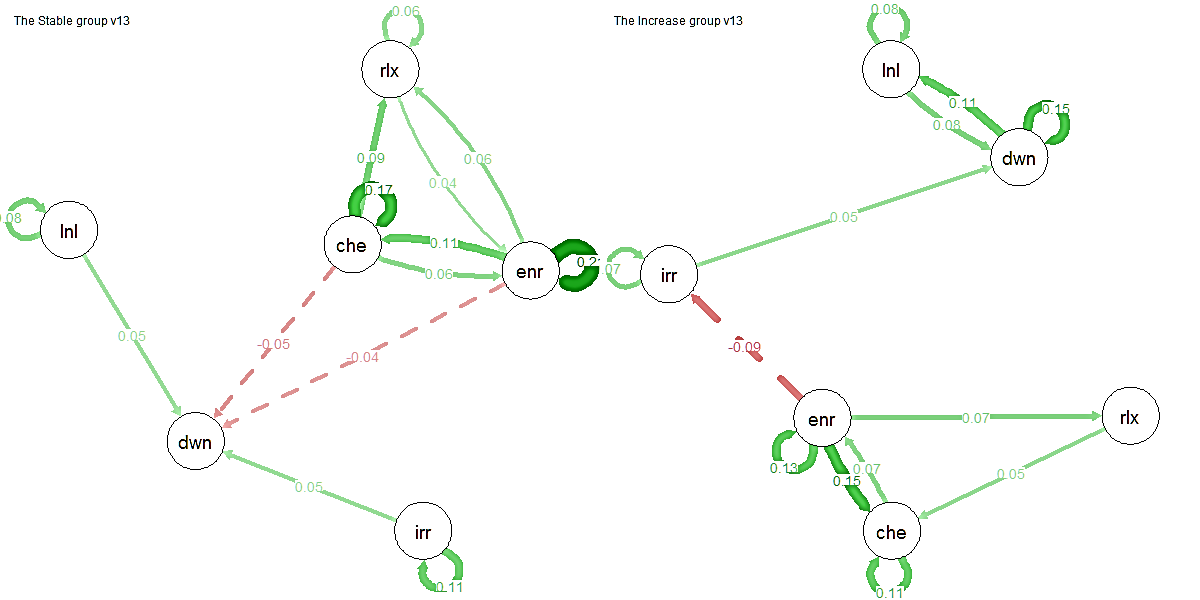 | 0 | 3 | 2 | 1 |
| 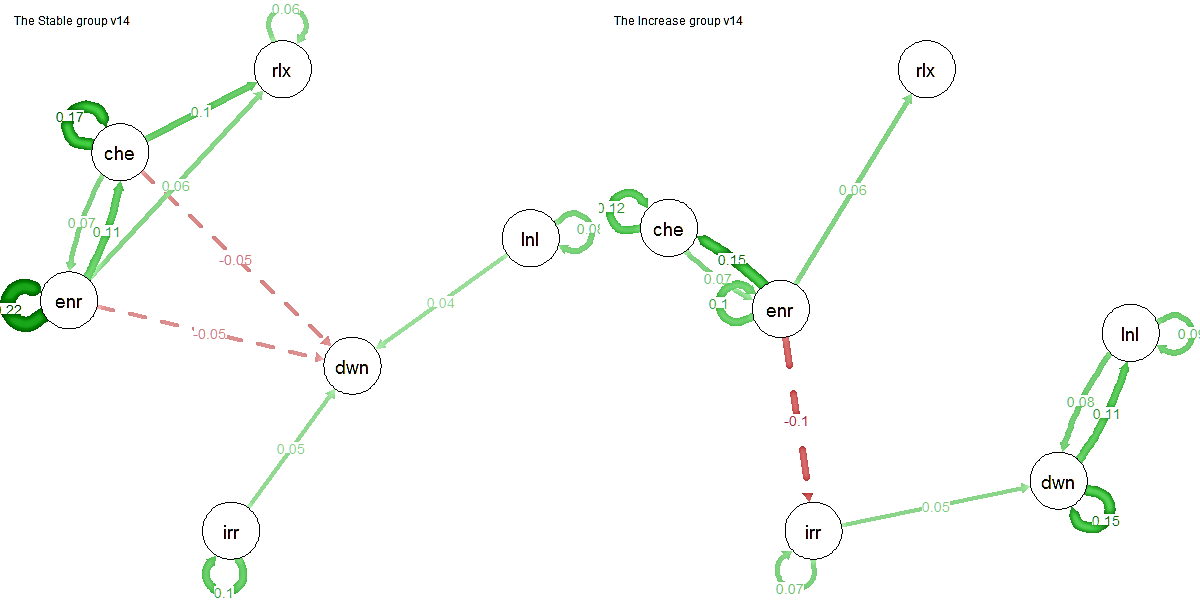 | 0 | 3 | 2 | 1 |
| 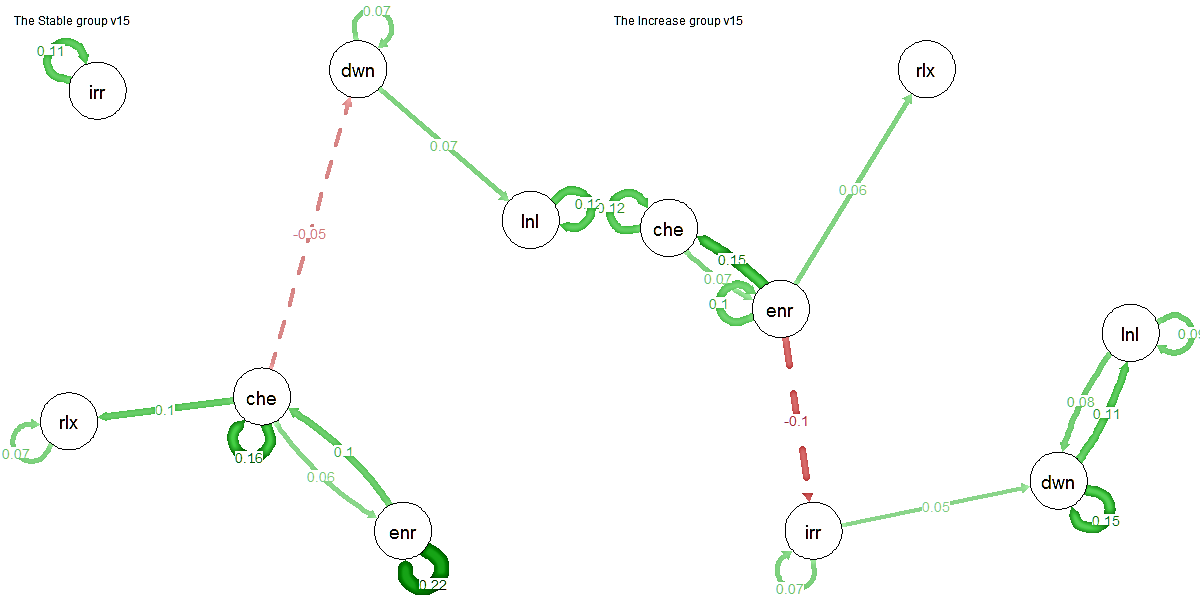 | 0 | 2 | 1 | 1 |
| 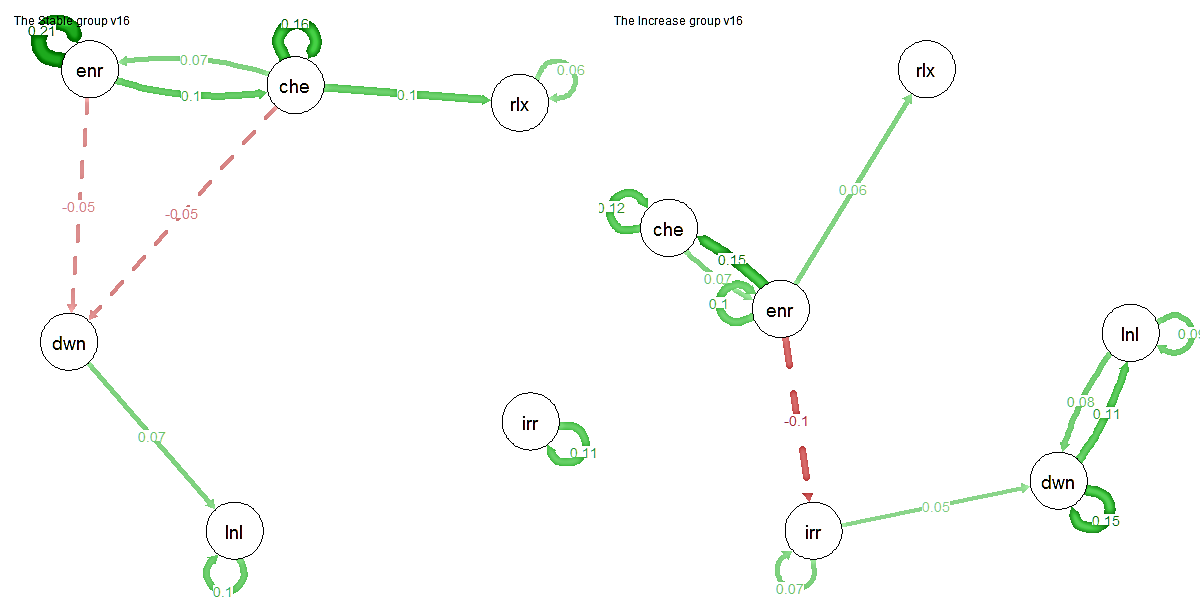 | 0 | 2 | 2 | 1 |
| 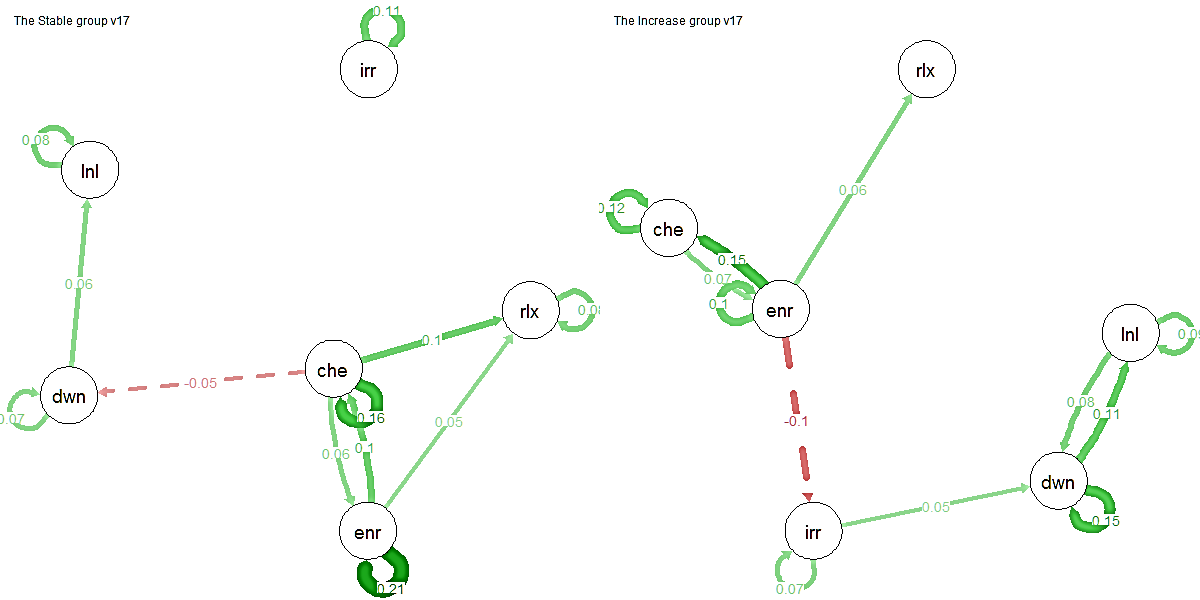 | 0 | 2 | 1 | 1 |
| 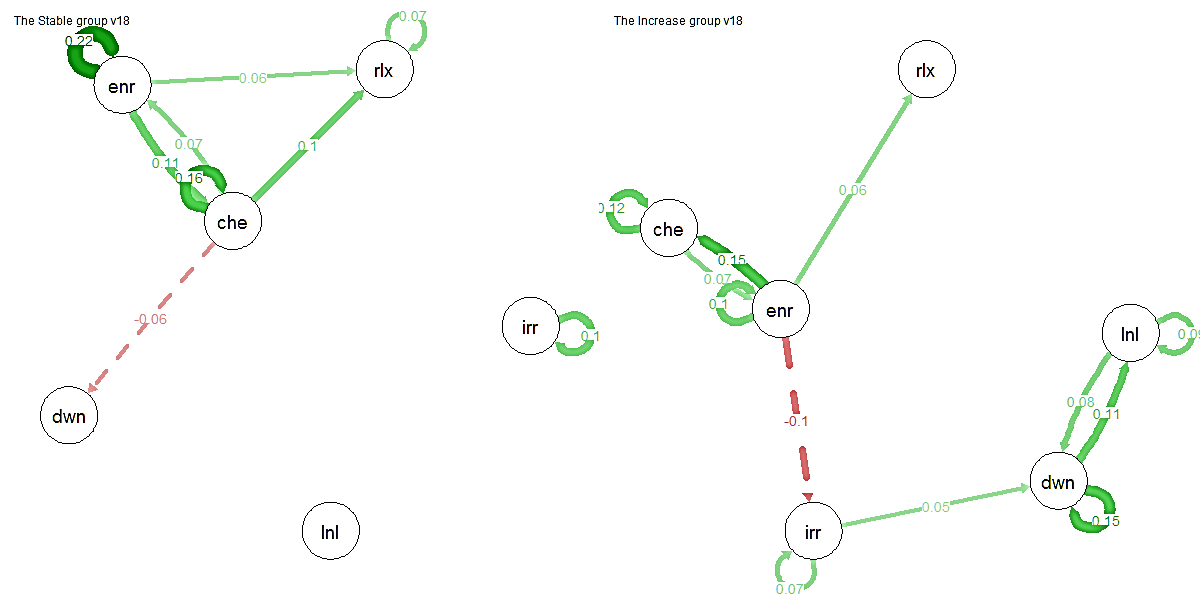 | 0 | 0 | 1 | 1 |
| 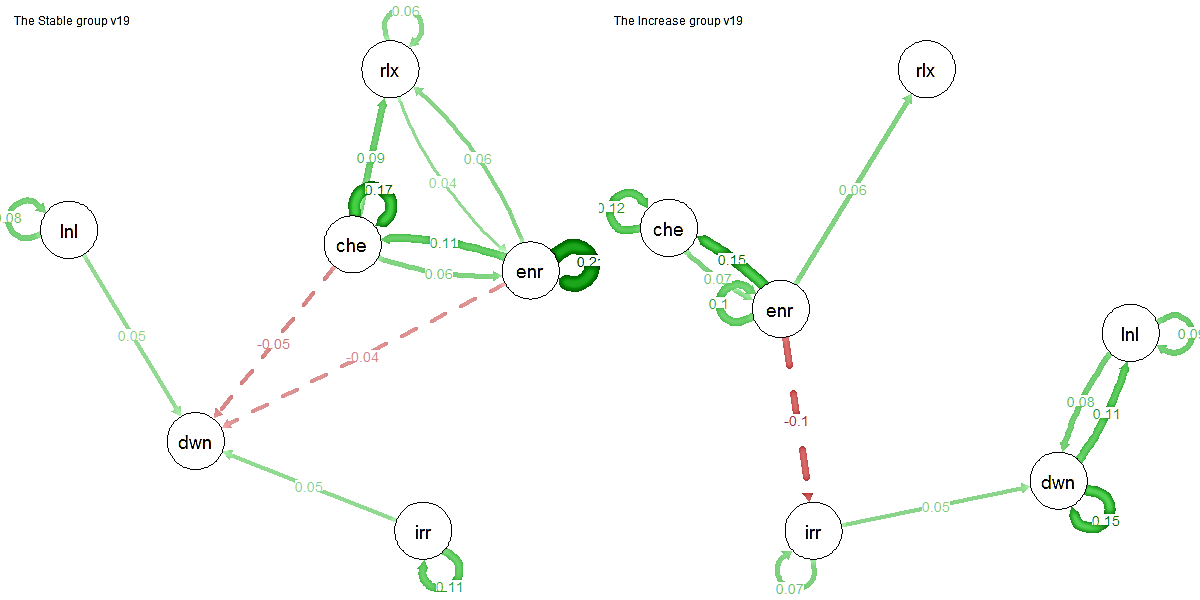 | 0 | 3 | 2 | 1 |
| 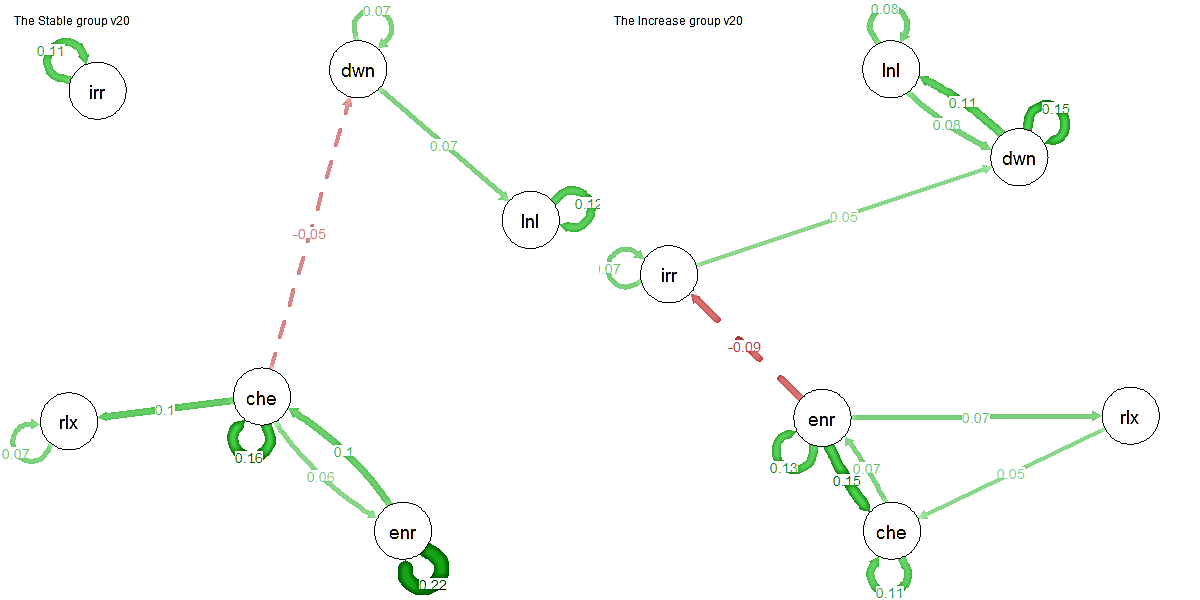 | 0 | 2 | 1 | 1 |
| 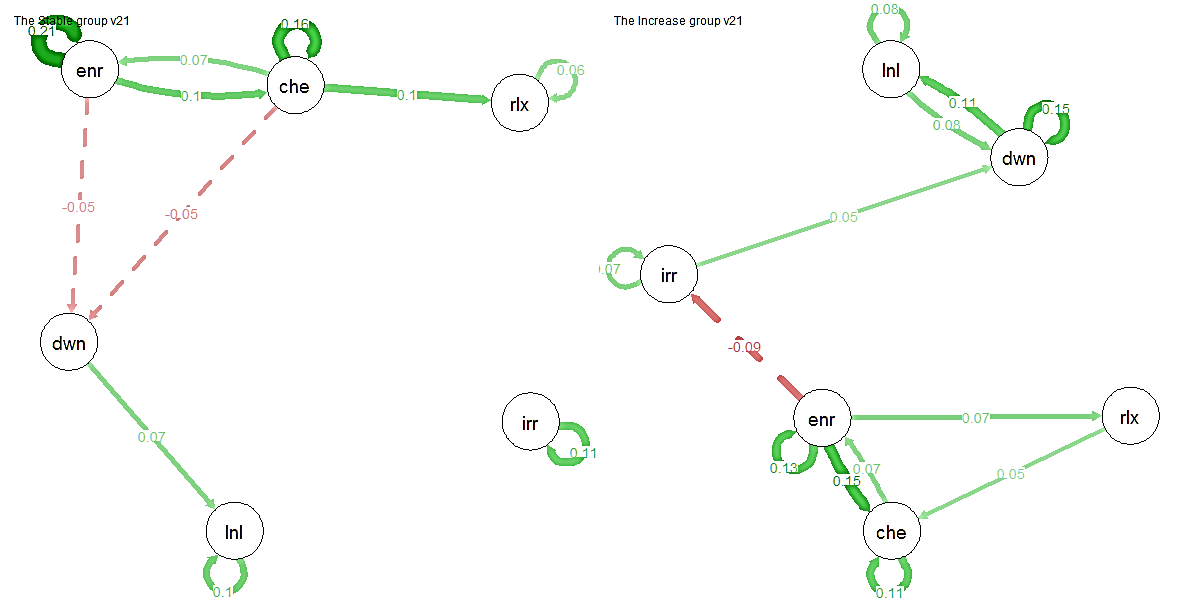 | 0 | 2 | 2 | 1 |
| 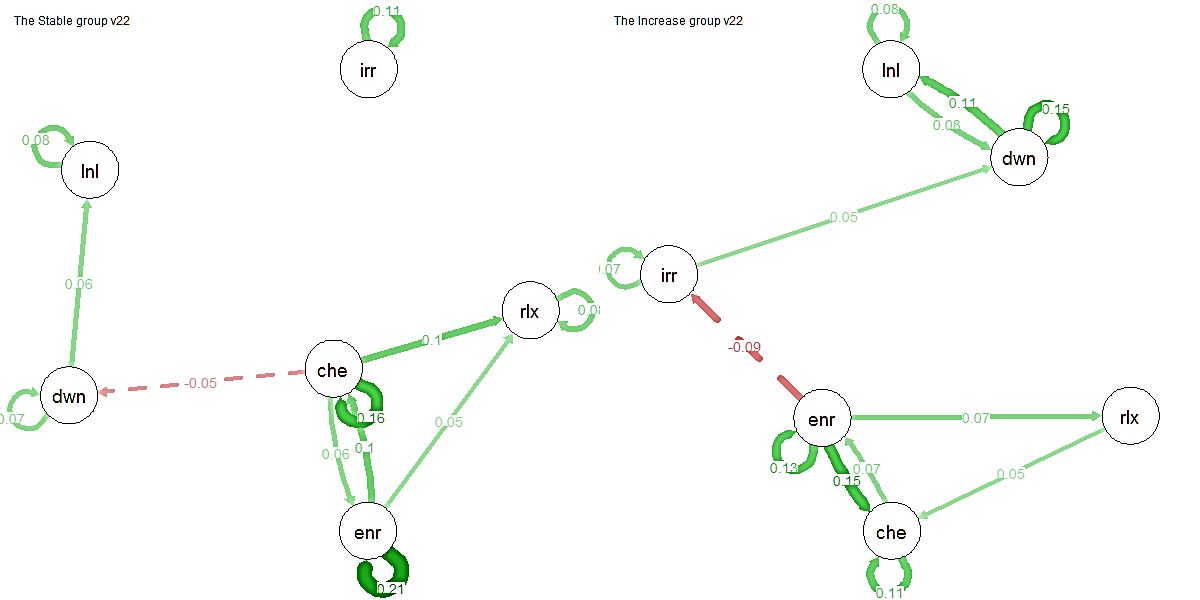 | 0 | 2 | 1 | 1 |
| 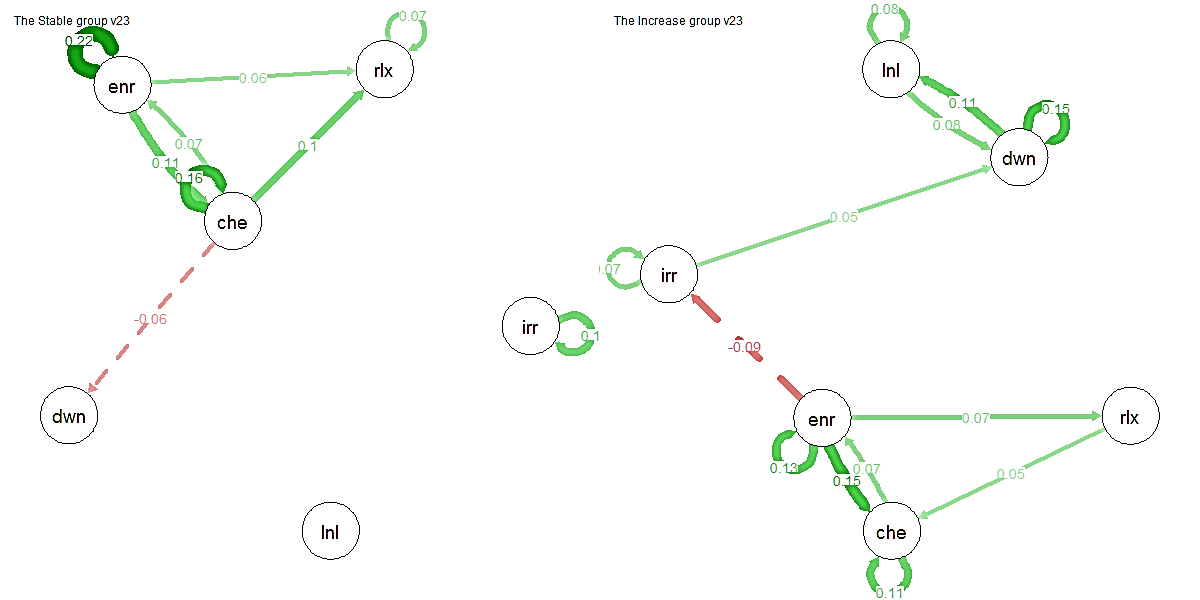 | 0 | 0 | 1 | 1 |
| 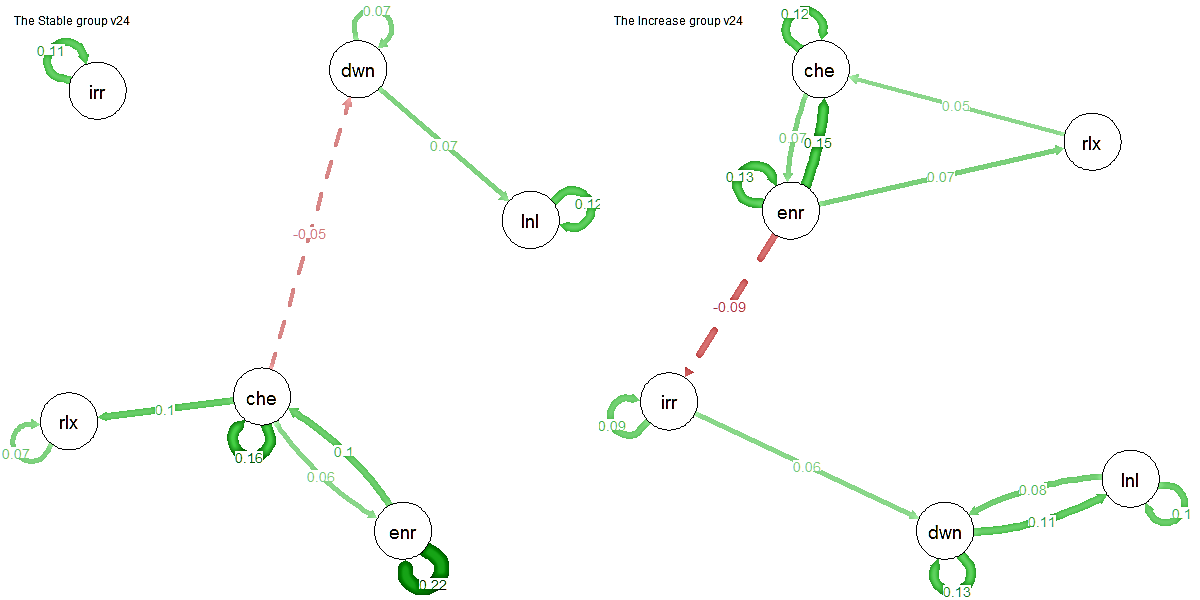 | 0 | 2 | 1 | 1 |
| 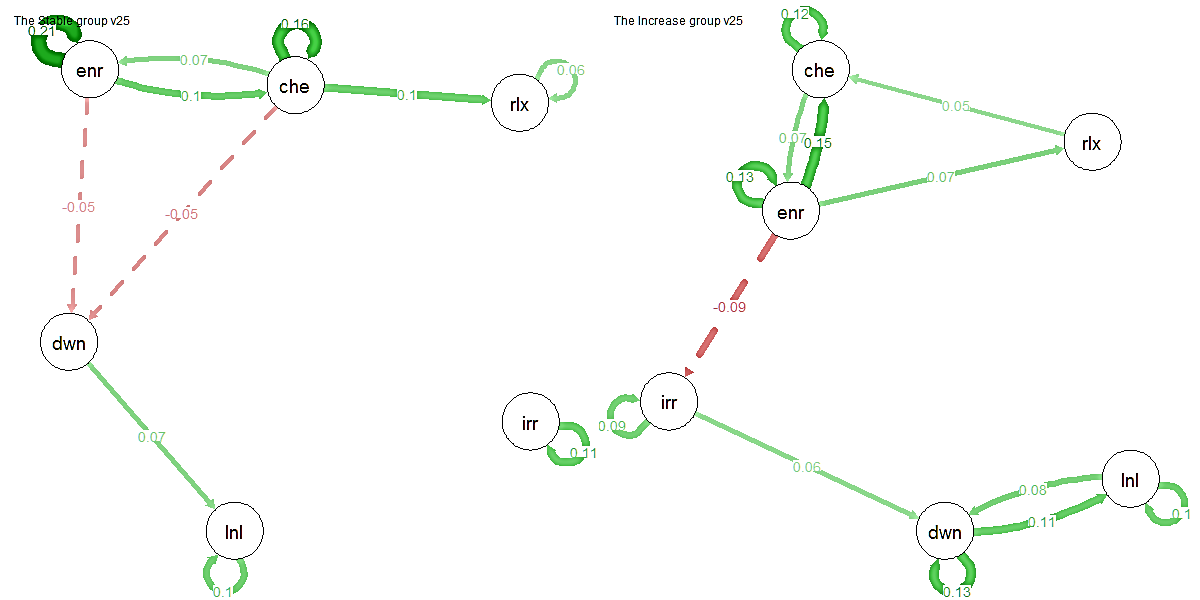 | 0 | 2 | 2 | 1 |
| 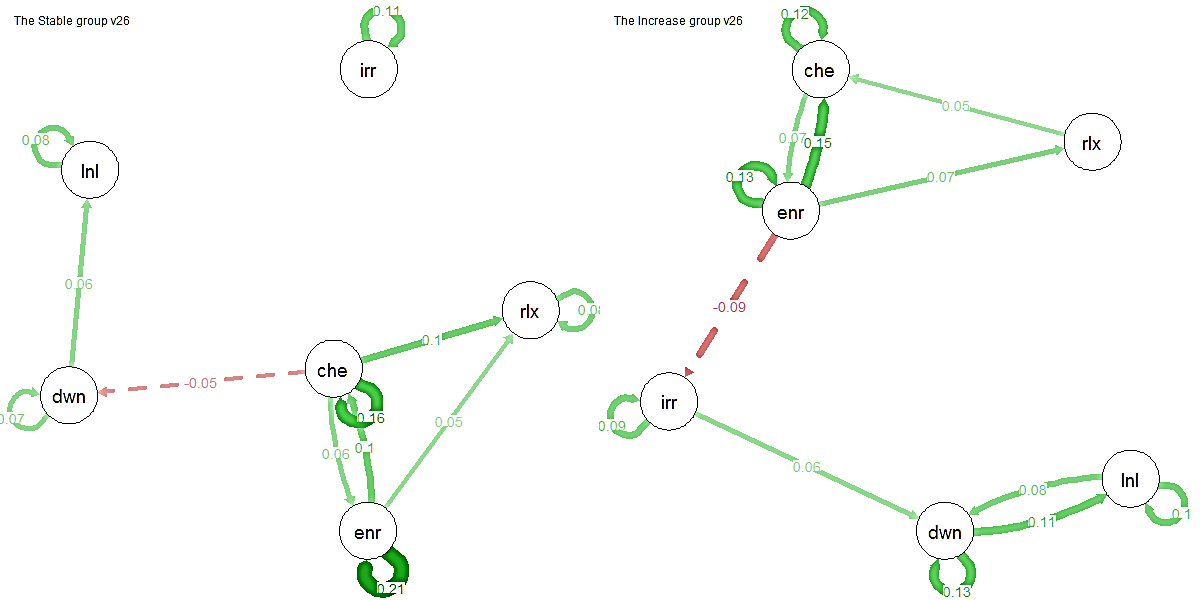 | 0 | 1 | 1 | 1 |
| 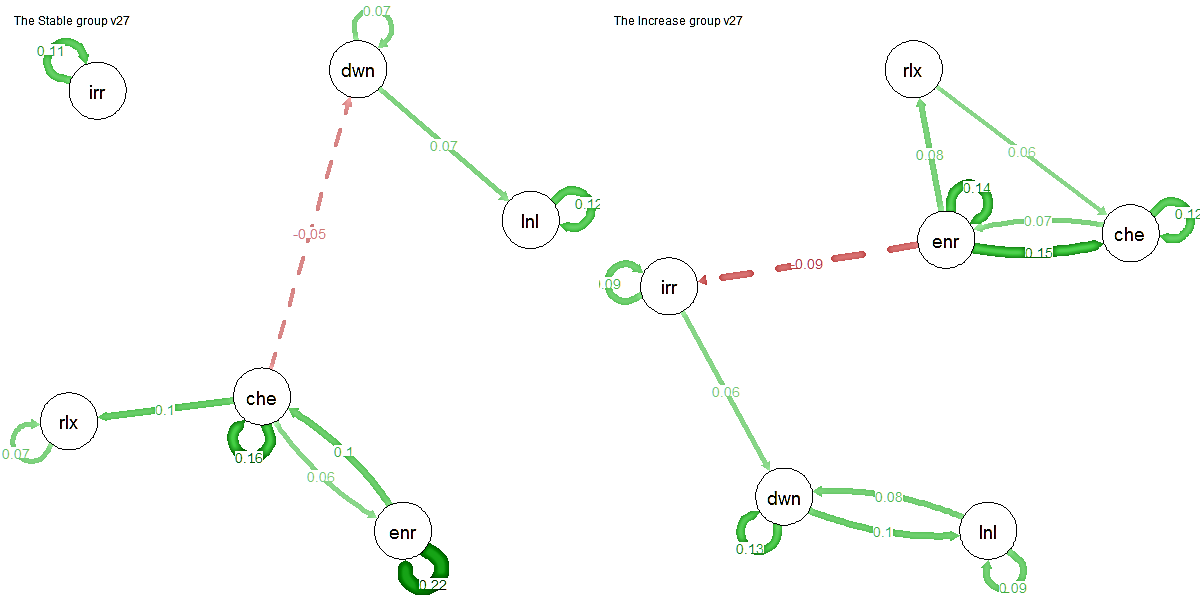 | 0 | 2 | 1 | 1 |
| 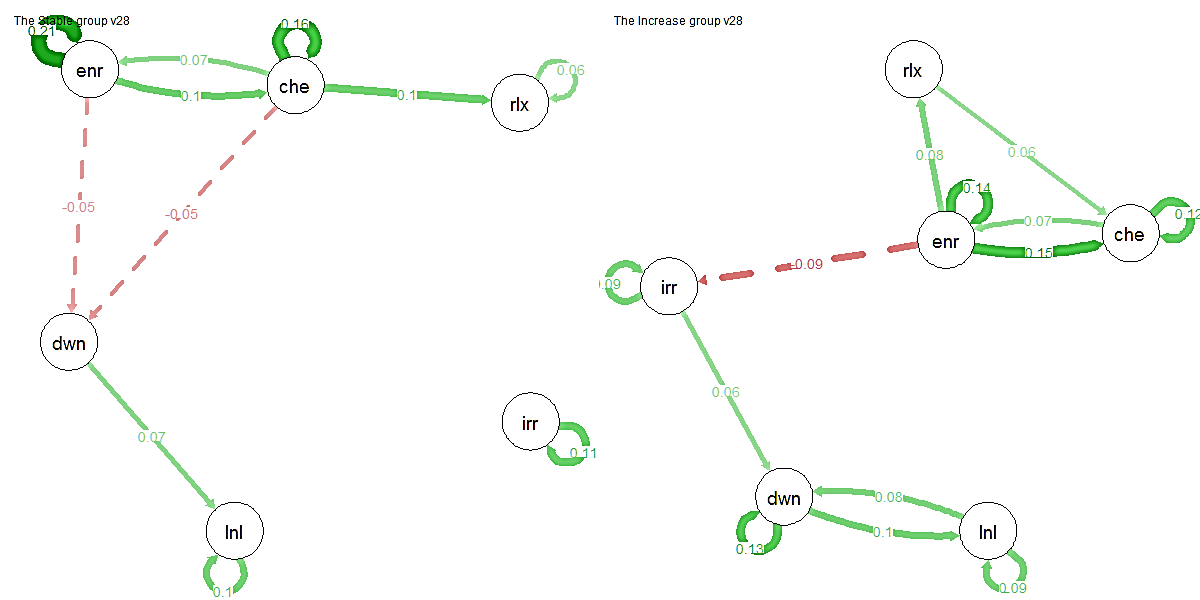 | 0 | 1 | 2 | 1 |
| 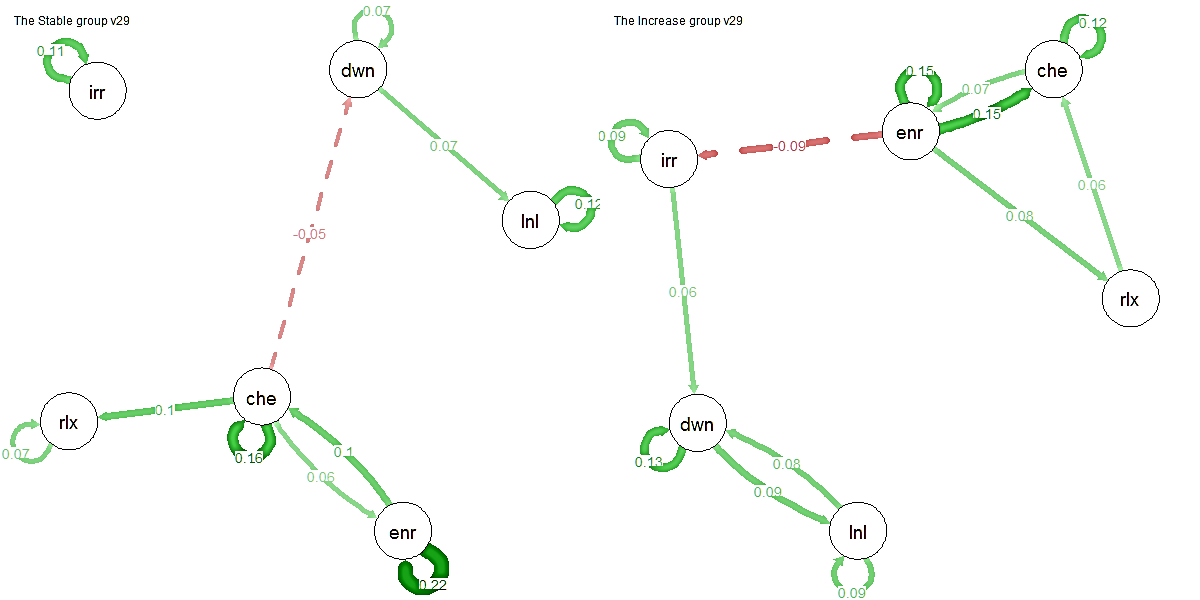 | 0 | 1 | 1 | 1 |
