# Supplementary material for: Network dynamics of momentary affect states and future course of psychopathology in adolescents
Source: PLoS One. 2021 Mar 4;16(3):e0247458. doi: 10.1371/journal.pone.0247458 (PMC7932519; doi:10.1371/journal.pone.0247458)
Supplement: S1 Text — (DOCX) [file pone.0247458.s004.docx]

### S1 Text. The detailed explanation of the calculations for the aims of the study

Our first aim was to examine whether the network of the Increase group had stronger connections between negative affect states than the network of the Stable group. For this aim, models were fitted with the original groups and the difference in negative connectivity between the groups was calculated using the permutation test. This was done by calculating the sum of all the connections between negative states (real values of all the possible paths *between* the nodes ‘irritated,' ‘down', and ‘lonely', excluding autocorrelations) for the Increase group and subtracting that from the sum of the connections between negative states for the Stable group. Thereafter, group labels (‘Stable’ or ‘Increase’) were randomly reassigned to the participants, and models were fitted again to those new random groups. This procedure was repeated 10,000 times; in this way, the permutation distribution of the possible group differences was created. After that, the observed group differences were compared to the permutation distribution to obtain p-values. A more detailed explanation of the procedure may be found elsewhere [1].

Our second aim was to investigate difference in influence of positive affect states in the networks. For this, we used two approaches. First, we evaluated out-degree centrality for all three positive nodes (‘Cheerful’, ‘Relaxed’, and ‘Energetic’) for the two groups separately. Out-degree centrality represents the strength of outward connections from this node to the others and was calculated as the sum of the absolute values of all outgoing connections (excluding autocorrelation) for the positive nodes in the network. Therefore, a high out-degree means that this node has more influence on other nodes in the network. For this comparison, we also used the permutation testing with the above described procedure, using the differences in the out-strengths of the positive affect states instead of negative connectivity (i.e., outdegree for 'cheerful' in the one group was compared with out-degree for ’cheerful’ for the other group, and so on), and tested these differences with the permutation test using 10,000 permutations. Second, we calculated the sum of all connection values from the positive affect states to the negative affect states, and vice versa. For that, we used the real values instead of the absolute values because we wanted to see only the suppressing effect: if some nodes upregulated the nodes with opposite modality, if would have cancelled out a part of the overall effect. Then these differences between groups were also tested with the same permutation procedure with 10,000 permutations.

Our last aim was to explore the dynamic structure of the networks in terms of the potential in the network to end up in possible vicious cycles. For this purpose, we visualized only the significant connections (based on the p-values, obtained from the multilevel mixed models) and evaluated the resulting structures of the networks visually.

1. Klippel A, Viechtbauer W, Reininghaus U, Wigman JTW, van Borkulo CD, Myin-Germeys I, et al. The Cascade of Stress: A Network Approach to Explore Differential Dynamics in Populations Varying in Risk for Psychosis. Schizophr Bull. 2017; 1–10. doi:10.1093/schbul/sbx037
