# Supplementary material for: Network dynamics of momentary affect states and future course of psychopathology in adolescents
Source: PLoS One. 2021 Mar 4;16(3):e0247458. doi: 10.1371/journal.pone.0247458 (PMC7932519; doi:10.1371/journal.pone.0247458)
Supplement: S2 Text — (DOCX) [file pone.0247458.s005.docx]

S2 Text. Limited multiverse analysis

In the current work, the group allocation was based on the tertiles of the change in SCL-90 symptoms. The reason behind such allocation was to use as many participants as possible, and came up with the groups with same starting level of symptoms and different future symptoms trajectories. However, the cut-off between groups and precise group allocation was somewhat subjective and depended on the algorithm used in the R-function (*xtile* from ‘stata’ package by M. Gomez, [link](https://cran.r-project.org/web/packages/statar/statar.pdf)). Therefore, there were many alternative possible group allocations with different cut-off scores.

Thus, to explore to what extend the cut-off for the SCL-90 scores for the creation of the different groups influences the results, we performed a limited multiverse analysis (based on Steegen et al., 2016 [2]), restricted only to different choices with regards to group allocation. Thus, we created groups all potential alternative “Stable” and “Increase” groups based on the different cutoff scores of SCL-90 change, with the following parameters: each group should have (i) at least 70 people (power restriction), (ii) comparable levels of SCL-90 scores and mean level of all six affect states at baseline, and (iii) different levels of SCL-90 scores at follow-up. After that, as the main findings of our study lie in visual assessment of the group networks (with only significant edges visualized), we have created such networks for all possible group combinations. For each network, we specified (i) the number of possible vicious cycles, (ii) the number of nodes in the negative “cluster”, (iii) number of downregulating paths from positive cluster to negative one or separate negative nodes.

Results: There were 29 possible group allocations. For all versions of “Increase groups” the network structure remained almost similar, with three interconnected negative nodes in one negative cluster comprising possibilities for “vicious circle”. The networks for “Stable” groups had more variations but (almost) all had the similar structure to the one reported in the main analysis and fitted the pattern of (almost) absence of “vicious” cycles, fewer negative clusters and connections and more downregulating connections from positive cluster to negative nodes. Specifically, among these networks, only one (~3.5%) contained the possibility for a “vicious cycle”; eight (~27.6%) networks of a “stable” groups contained upregulating connections between any 3 negative nodes (without forming self-reinforcing loops, i.e. two connections and three nodes, e.g. from “Lonely” to “Down” and from “Irritated” to “Down”), 14 (~48.3%) networks of a “stable” groups contained upregulating connections between any 2 negative nodes (i.e. one connection between two nodes, e.g. from “Lonely” to “Down”; with the exception of the network with “vicious cycle” containing two connections between two negative nodes), and seven (~24.1%) contained no connections between negative nodes and therefore no negative cluster at all. Moreover, all networks of both groups contained downregulating connections from a positive cluster to at least one negative node but among 29 networks of “stable” groups, 11 (~37.9%) contained two downregulating connections from positive cluster to negative nodes, whereas all the networks of “increase” groups contained only one such connection. The table S3 shows the combinations of group networks and their characteristics. Because all networks of “Increase” group were similar, only characteristics of “Stable” groups are presented in the table s3.

2. Steegen S, Tuerlinckx F, Gelman A, Vanpaemel W. Increasing Transparency Through a Multiverse Analysis. Perspect Psychol Sci. 2016. doi:10.1177/1745691616658637
