# Supplementary figures and images for: Network dynamics of momentary affect states and future course of psychopathology in adolescents
Source: PLoS One. 2021 Mar 4;16(3):e0247458. doi: 10.1371/journal.pone.0247458 (PMC7932519; doi:10.1371/journal.pone.0247458)

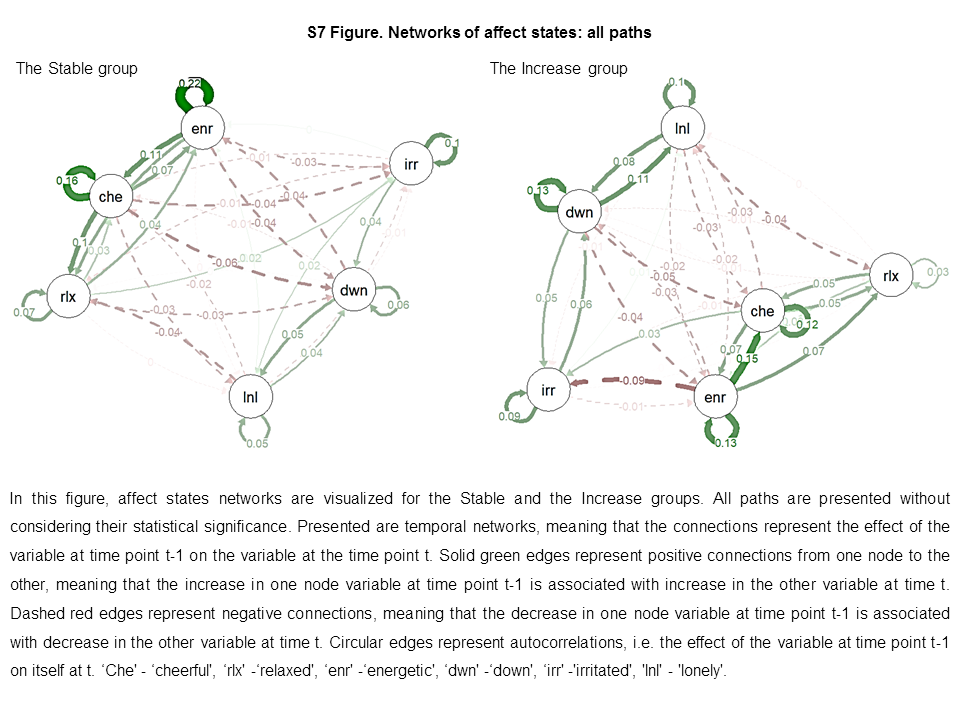

Supplement: S1 Fig — In this figure, affect states networks are visualized for the Stable and the Increase groups. All paths are presented without considering their statistical significance. Presented are temporal networks, meaning that the connections represent the effect of the variable at time point t-1 on the variable at the time point t. Solid green edges represent positive connections from one node to the other, meaning that the increase in one node variable at time point t-1 is associated with increase in the other variable at time t. Dashed red edges represent negative connections, meaning that the decrease in one node variable at time point t-1 is associated with decrease in the other variable at time t. Circular edges represent autocorrelations, i.e. the effect of the variable at time point t-l on itself at t. ’Che’—’cheerful’, ’rlx’ -’relaxed, ’enr’ -’energetic’, ’dwn’ -’down’, ’irr’ -’irritated’, ’lnl’ -lonely’. (TIF) [file pone.0247458.s007.tif]
